# The potential of adipokines in identifying multiple trauma patients at risk of developing multiple organ dysfunction syndrome

**DOI:** 10.1186/s40001-021-00511-z

**Published:** 2021-04-30

**Authors:** Julian Haupt, Niels Krysiak, Marina Unger, Viktoria Bogner-Flatz, Peter Biberthaler, Marc Hanschen, Martijn van Griensven, Alexander T. Haug

**Affiliations:** 1 Experimental Trauma Surgery, Klinikum rechts der Isar, Technical University of Munich, Ismaninger Strasse 22, 81675 Munich, Germany; 2 Bundeswehr Institute of Radiobiology affiliated to the University Ulm, Neuherbergstrasse 11, 80937 Munich, Germany; 3 Department of Trauma Surgery, University Hospital Munich, Ludwig-Maximilians-University, Nussbaumstrasse 20, 80336 Munich, Germany; 4 Department of Trauma Surgery, Klinikum rechts der Isar, Technical University of Munich, Ismaninger Strasse 22, 81675 Munich, Germany; 5 Department of Cell Biology-Inspired Tissue Engineering (cBITE), MERLN Institute for Technology-Inspired Regenerative Medicine, Maastricht University, Universiteitssingel 40, 6229 ER Maastricht, The Netherlands; 6 Department of Orthopedics and Sports Orthopedics, Klinikum rechts der Isar, Technical University of Munich, Ismaninger Strasse 22, 81675 Munich, Germany

**Keywords:** Multiple trauma, Multiple organ dysfunction syndrome, Multiple organ failure, Adipokines, Leptin, Resistin, Interleukin-17A, Interleukin-33

## Abstract

**Background:**

Multiple organ dysfunction syndrome (MODS) and the consecutive multiple organ failure (MOF) are severe and dreaded complications with a high mortality in multiple trauma patients. The aim of this study was to investigate the potential of the adipokines leptin, resistin, interleukin-17A and interleukin-33 as possible biomarkers in the early posttraumatic inflammatory response and for identifying severely traumatized patients at risk of developing MODS.

**Methods:**

In total, 14 multiple trauma patients with an injury severity score (ISS) ≥ 16 as well as a control group of 14 non-multiple trauma patients were included in this study and blood samples were taken at the time points 0, 6, 24, 48 and 72 h after admission. For the trauma patients, the SIRS and Denver MOF score were determined daily. The quantitative measurement of the plasma concentrations of the adipokines was performed using ELISA.

**Results:**

In the statistical analysis, the multiple trauma patients showed statistically significant higher plasma concentrations of leptin, resistin, IL-17A and IL-33 compared to the control group. In addition, there was a statistically significant positive correlation between the concentrations of resistin, IL-17A and IL-33 and the corresponding SIRS scores and between the concentrations of resistin, IL-17A and IL-33 and the corresponding Denver MOF scores. Finally, ROC curve analysis revealed that the adipokines leptin and IL-17A are suitable diagnostic markers for the discrimination between multiple trauma patients with and without MOF.

**Conclusions:**

Leptin and IL-17A could be suitable diagnostic markers to identify severely injured patients with a developing SIRS and MOF earlier, to adjust surgical therapy planning and intensive care.

## Background

Trauma represents the leading cause of death for people up to the age of 40 years in Germany [1, 2]. While overall mortality caused by severe trauma has decreased over the years, multiple organ dysfunction syndrome (MODS) and the consecutive multiple organ failure (MOF) are still severe complications with a high mortality [3–5]. MODS is caused by an imbalance or a dysregulation of the pro- and anti-inflammatory immune response [6, 7]. To identify MOF, the Denver multiple organ failure score may be used, which detects a dysfunction of the lung, kidneys, liver and heart. The dysfunctions of the respective organ systems are categorized into grade 0 to 3 and added to a final score between 0 and 12 points. A value > 3, which is calculated 48 h after the initial trauma, is defined as multiple organ failure [8–10].

Characteristic for the pro-inflammatory immune response, the systemic inflammatory response syndrome (SIRS), is a local and systemic production of various mediators such as pro-inflammatory cytokines, complement factors, acute phase proteins and an accumulation of immune cells at the site of inflammation [6]. To diagnose an SIRS, the SIRS score is used, which includes the parameters body temperature (< 36° or > 38°), heart rate (> 90 beats/min), respiratory rate (> 20 breaths/min) and the white blood cell count (< 4000/mm^3^ or > 12,000/mm^3^). A point can be assigned for each abnormality in one of these four parameters, so that the score can have a value between 0 and 4 and is positive for a value ≥ 2 [11].

Adipokines are bioactive proteins that are produced by adipocytes and cells of the immune system [12, 13]. Adipokines exert their effect on metabolism, immunity and inflammation via an endocrine, paracrine and autocrine secretion mode [14].

The adipokine leptin is induced by TNF-α via the p55 TNF receptor in adipocytes and is a pro-inflammatory acute phase protein and a regulator of the fat metabolism [12, 15–17]. Leptin exerts its pro-inflammatory properties via an activation and proliferation of monocytes and an increase in the production of TNF and IL-6 in the activated monocytes [12, 16, 18]. Leptin also induces chemotaxis and liberation of oxygen radicals from neutrophilic granulocytes [16]. On the other hand, leptin decreased mortality in traumatized and septic wild-type mice [19]. The pro-inflammatory adipokine resistin is synthesized and secreted by adipocytes, endothelial cells, bone marrow cells and especially by mononuclear cells [20, 21]. The synthesis is induced by the pro-inflammatory cytokines IL-1, IL-6 and TNF-α as well as by lipopolysaccharides [22]. Resistin mediates its pro-inflammatory effect via the NF-κB signaling pathway and induces the synthesis of the pro-inflammatory cytokines IL-1β, IL-6 and TNF-α in mononuclear cells [23].

The adipokine IL-17A is mainly secreted by TH-17 cells and is induced by the pro-inflammatory cytokines IL-1, IL-6, IL-23 and TGF-β [24–26]. IL-17A is a potent activator of neutrophil granulocytes and promotes their recruitment and migration [25, 26]. IL-17A also activates T and B cells, promotes priming of T cells and antibody production as well as antibody class changes in B cells [24, 27].

Interleukin-33 is expressed by a variety of cell types, including adipocytes, endothelial cells, fibroblasts and macrophages [28, 29]. The release of IL-33 occurs mainly in the context of necrosis of cells, for example, in a trauma or an infection and thus acts as an alarmin [30]. IL-33 acts as a chemoattractant and induces the production of the cytokines IL-5 and IL-13 in type 2 T helper cells [28, 31]. In addition, IL-33 leads to the activation of eosinophilic granulocytes with an increased production of superoxide anions and IL-8, an induction of degranulation and an increase in cell survival [32].

To classify the severity of trauma or multiple trauma, the Abbreviated Injury Scale (AIS) and the Injury Severity Score (ISS) are used. The AIS describes unique characteristics, severity and lethality of a single injury and is composed of a seven-digit code. The seventh and last digit denominates the severity of the injury on a scale from 1 to 6, with the number 6 representing a lethal injury [33, 34]. The seventh digit ultimately forms the basis for calculating the ISS, which was first published in 1974 by Baker et al. and is a scoring system for assessing the severity of injury and the lethality of trauma patients. The severity of the injury is determined according to the AIS with a number from 1 to 6 for six body regions (head/neck, face, chest, abdomen, extremities, external) and the points of the three most severely injured body regions are squared and summed to the final ISS between 0 and 75 points [35, 36].

Since the role of the adipokines leptin, resistin, interleukin-17A and interleukin-33 in trauma immunology has been poorly understood, the aim of this pilot study is to investigate the response of adipokines in the early posttraumatic systemic inflammatory immune response and whether adipokines are suitable diagnostic biomarkers to identify multiple trauma patients at risk of developing MODS in an intensive care setting.

## Methods

### Patients

The study was performed at our level 1 trauma center in accordance with the Good Clinical Practice Guidelines and the ethical standards as laid down in the 1964 Declaration of Helsinki and its later amendments. Ethical committee approval was obtained from the local institutional review board of the University of Munich (reference number: 012/00). Signed informed consent was obtained from the patients or legal guardians. In this study, we included multiple trauma patients aged 18 and older with an injury severity score (ISS) of ≥ 16. A total of 14 patients between the age of 19 and 79 years (mean: 49.7 ± 19.2 years) were enrolled in our 1-year inclusion period. The patient population consisted of six women and eight men. As a control group, we included 14 non-multiply traumatized individuals, nine of whom suffered a simple fracture on one extremity (humerus, forearm or lower leg) from a monotrauma and five were completely healthy. The nine patients with the fracture received a single plate osteosynthesis. The control group consisted of eight women and six men between the age of 23 and 60 years (41.2 ± 12.2 years). Exclusion criteria for the multiple trauma and the control group were a malignant or infectious disease, patients under immunosuppressive therapy and pregnancy. Furthermore, individuals in the control group with an abnormal routine organ blood profile were also excluded.

### Blood samples

Four S-Monovettes EDTA 9 ml (Sarstedt, Nümbrecht, Germany) blood samples were drawn from the multiple trauma patients on admission (not later than 90 min after trauma, indicated as 0 h) and after 6, 24, 48 and 72 hours (h) at our intensive care unit. In total, 63 different blood samples could be obtained from the multiple trauma patients. The 7 missing samples are due to the fact that at point of time 0 h two of the patients were unstable or had to be resuscitated, and therefore, no blood was drawn in order not to endanger the patient's health and two of the patients died before the end of the observation period. From the control group, one S-Monovette EDTA 9 ml (Sarstedt, Nümbrecht, Germany) blood sample was drawn. The blood sample from the patients with a fracture was drawn 24 h after the osteosynthesis and the blood sample from the healthy participants during the day. The EDTA S-Monovettes were centrifuged for 10 min at 350×*g* and a temperature of 20 °C (Centrifuge 5810 R, Eppendorf, Hamburg, Germany). After the centrifugation, 750 μl plasma was transferred to Eppendorf Safe-Lock Tubes (1.5 ml) and stored at – 80 °C until further processing and testing.

### Clinical parameters/scores

For each multiple trauma patient, the injury severity score (ISS) was calculated based on clinical findings, radiological examinations and intraoperative findings [35, 36]. In addition, the SIRS and Denver MOF score were calculated on a daily basis.

To determine the Denver MOF Score, the ratio PaO_2_/FiO_2_, creatinine (μmol/l), total bilirubin (μmol/l) and an inotropic medication were recorded.

### Enzyme-Linked Immunosorbent Assay (ELISA)

The quantitative measurement of the adipokine plasma concentrations was performed with the following commercially available Enzyme-Linked Immunosorbent Assays (PeproTech, Hamburg, Germany): Human Leptin ELISA Development Kit (Catalog number: 900-K90/Lot number: 0710090); Human Resistin Mini ELISA Development Kit (Catalog number: 900-M235/Lot number: 0412235); Human IL-17A Mini ELISA Development Kit (Catalog number: 900-M84/Lot number: 1212084-M); Human IL-33 Mini ELISA Development Kit (Catalog number: 900-M398/Lot number: 1112398-M). All ELISAs were performed according to the manufacturer’s manuals. The photometric measurement was performed with the ELISA Plate Reader FLUOstar Omega and the Reader Control Software (BMG Labtech, Ortenberg, Germany).

### Statistics

The calculation of the adipokine concentrations from the OD values of the ELISAs, the statistical analysis of the data and the creation of the diagrams was performed with GraphPad PRISM Version 8.0.1 (GraphPad Software Inc., La Jolla, USA). The calculation of the individual adipokine concentrations from the OD values was carried out by an interpolation of the concentrations from the generated standard curves. A normal distribution or logarithmic normal distribution of the data was tested with the D'Agostino–Pearson test (Omnibus K2). Comparisons between non-normally distributed values were analyzed with the non-parametric Mann–Whitney *U* test. Assuming non-normally distributed values, the analysis of a correlation between the adipokine concentrations of the polytrauma patients at the respective points in time and the ISS, SIRS score and the Denver MOF score was performed ​​with the non-parametric Spearman correlation. The specified correlation coefficient r, quantifies the direction and magnitude of correlation. The applicability of adipokines as diagnostic markers for the discrimination between multiple trauma patients with and without MOF was analyzed with receiver operating characteristic curves (ROC curves). The resulting area under a ROC curve (AUC) quantifies the overall ability to discriminate between those individuals with the disease and those without the disease.

A significance level α of 0.05 was specified and the results were considered statistically significant at a *p* value of < 0.05. The statistical analysis was checked for correctness by the Department of Statistics, Technical University of Munich.

## Results

### Clinical parameters

The values of the ISS of the individual multiple trauma patients ranged from 17 to 50, the median was 41. Of all included patients, 85.71% suffered a head injury (AIS ≥ 3), 71.42% a chest trauma (AIS ≥ 3) and 28.57% an abdominal trauma (AIS ≥ 3). All 14 multiple trauma patients developed SIRS within 72 h after trauma, with an interindividual distinct degree of severity. On the first day, 100% of all multiple trauma patients fulfilled the criteria for SIRS, on the second day 46.15% and on the third and fourth day 50%. In total, four patients suffered MOF according to the Denver MOF score with a value of > 3 within 3 days after trauma. Three of those four patients had a maximum MOF score of 4 and one patient a maximum score of 5. All of our multiple organ failure patients developed a failure of their lung and heart. The heart and lung failure was the leading and most severe failure in all of our patients. Nine patients including all the patients with MOF, died from their trauma sequelae within 18 days.

### Leptin

**Fig. 1:**
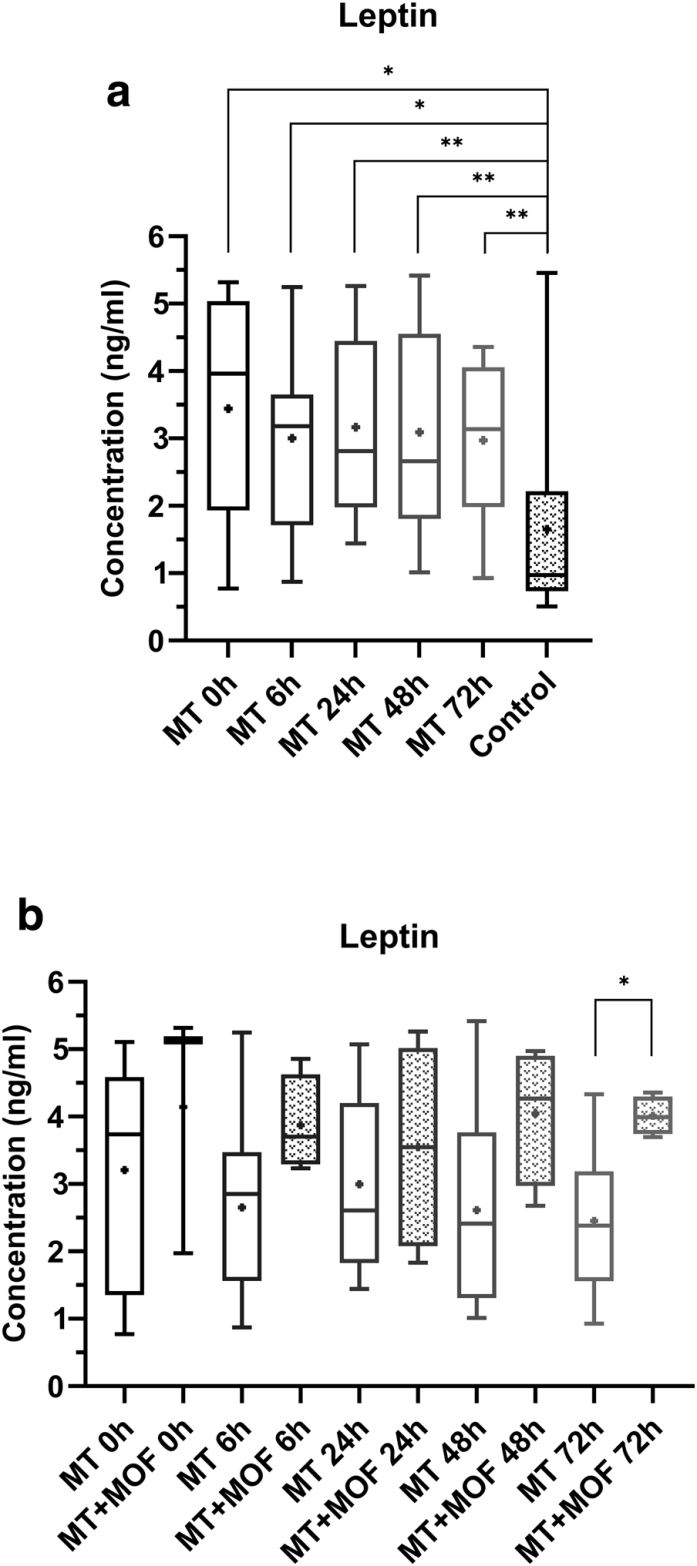
**a** Comparison of leptin plasma concentrations at the individual time points between multiple trauma patients (MT) and control group (Control). Depiction of the concentrations as box-and-whisker plots with minimum/maximum value and + depicting the mean value. Statistical analysis of the data was performed by Mann–Whitney *U* test (* = *p* < 0.05; ** = *p* < 0.01). n_multiple trauma 0 h = 12_, n_multiple trauma 6 h = 14_, n_multiple trauma 24 h = 13_, n_multiple trauma 48 h = 12_, n_multiple trauma 72 h = 12_, n_control group = 14_**. b** Comparison of leptin plasma concentrations at the individual time points between multiple trauma patients without (MT) and multiple trauma patients with multiple organ failure (MT + MOF). Depiction of the concentrations as box-and-whisker plots with minimum/maximum value and + depicting the mean value. Statistical analysis of the data was performed by Mann–Whitney *U* test (* = *p* < 0.05). n_MT 0 h = 9_, n_MT + MOF 0 h = 3_, n_MT 6 h = 10_, n_MT + MOF 6 h = 4_, n_MT 24 h = 9_, n_MT + MOF 24 h = 4_, n_MT 48 h = 8_, n_MT + MOF 48 h = 4_, n_MT 72 h = 8_, n_MT + MOF 72 h = 4_

The leptin plasma concentrations of the multiple trauma patients were statistically significant higher at time point 0 h (*p* value = 0.0148), 6 h (*p* value = 0.0141), 24 h (*p* value = 0.0023), 48 h (*p* value = 0.0077) and 72 h (*p* value = 0.0077), as compared to the control group (Fig. 1a). The multiple trauma patients with MOF showed higher leptin concentrations at all five time points and at 72 h, a statistically significant (*p* value = 0.0283) higher concentration compared to the multiple trauma patients without MOF (Fig. 1b).

**Fig. 2:**
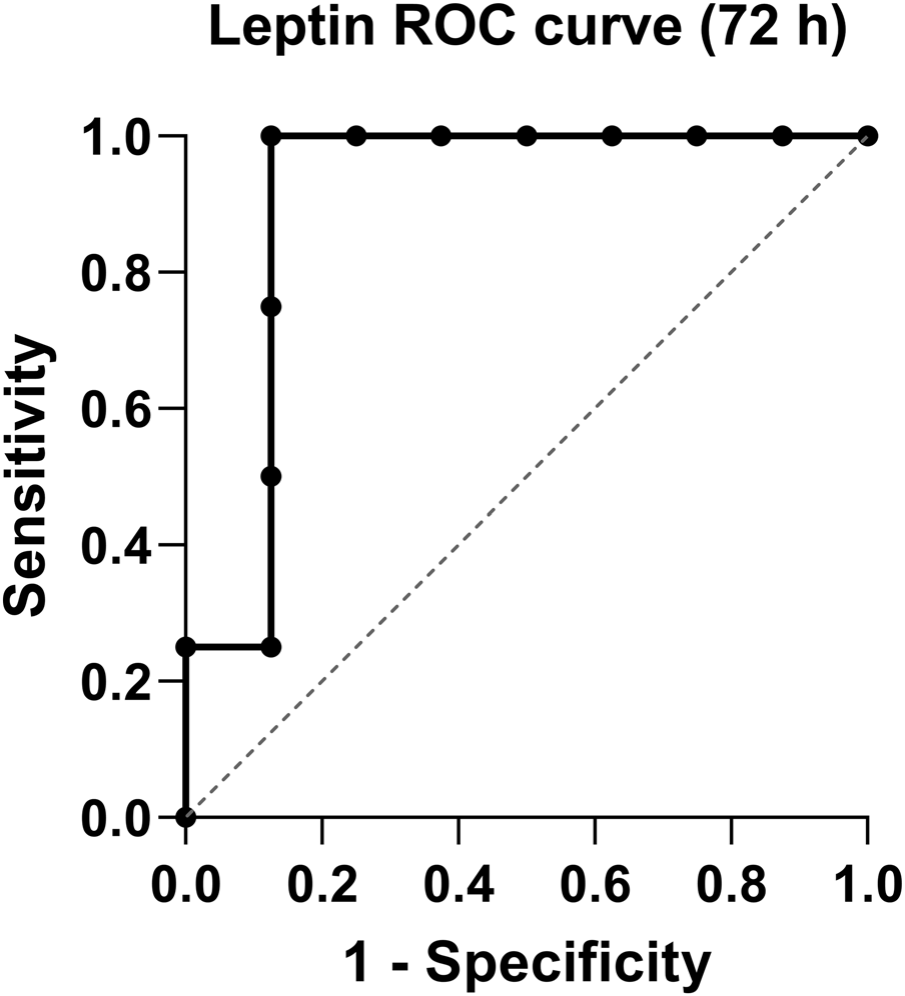
Receiver operating characteristic curve (ROC curve) analysis of leptin plasma concentration at time point 72 h for the discrimination between multiple trauma patients with and without multiple organ failure (AUC = 0.9063; 95% CI 0.7197–1.00; *p* = 0.0272)

The leptin concentration at the time point 72 h showed an area under the curve (AUC) of 0.9063 (95% CI  0.7197–1.00) and a statistical significance with a *p* value of 0.0272 (Fig. 2).

### Resistin

**Fig. 3:**
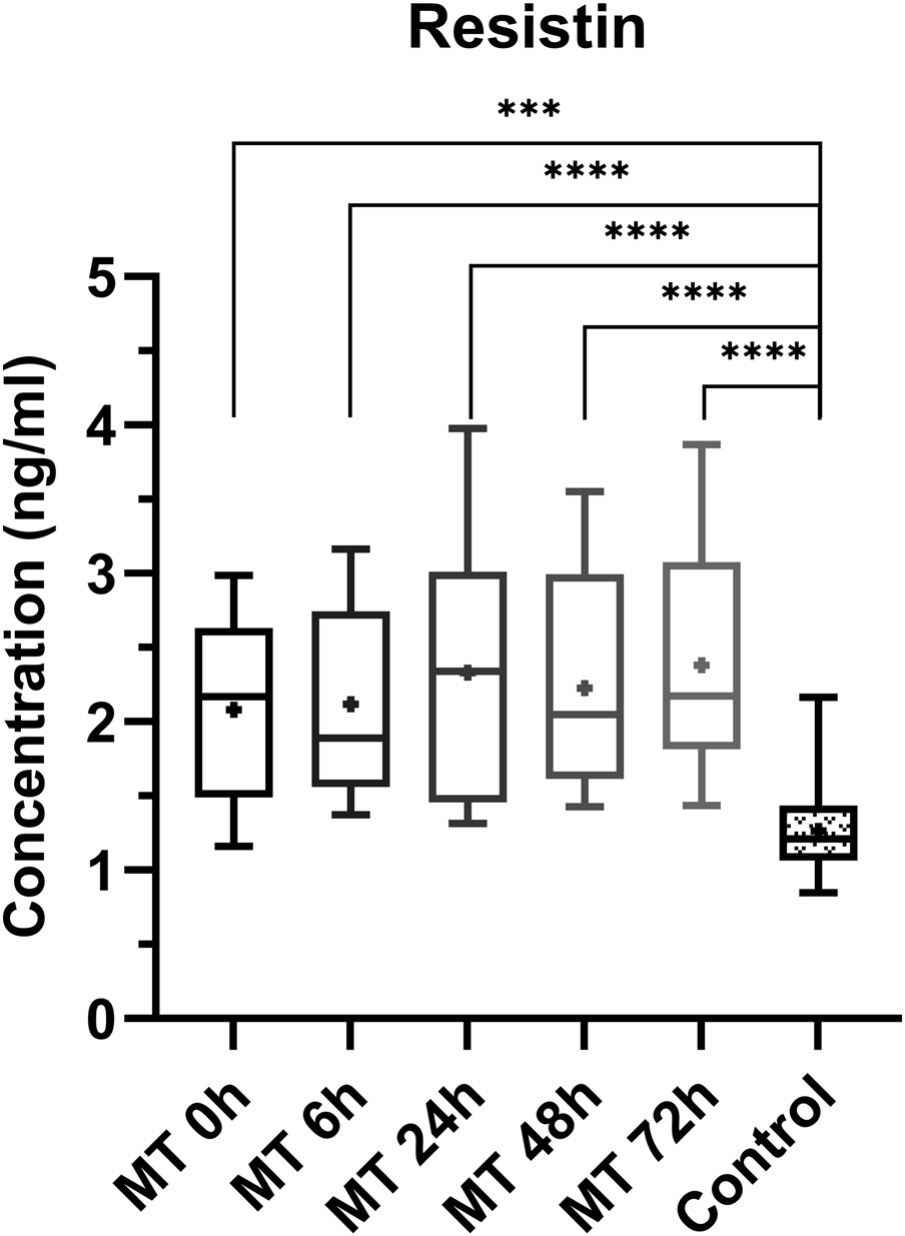
Comparison of resistin plasma concentrations at the individual time points between multiple trauma patients (MT) and control group (Control).Depiction of the concentrations as box-and-whisker plots with minimum/maximum value and + depicting the mean value. Statistical analysis of the data was performed by Mann–Whitney *U* test (*** = *p* < 0.001; **** = *p* ≤ 0.0001). n_multiple trauma 0 h = 12_, n_multiple trauma 6 h = 14_, n_multiple trauma 24 h = 13_, n_multiple trauma 48 h = 12_, n_multiple trauma 72 h = 12_, n_control group = 14_

The resistin plasma concentrations of the multiple trauma patients were statistically significant higher at time point 0 h (*p* value = 0.0004), 6 h (*p* value =  ≤ 0.0001), 24 h (*p* value =  ≤ 0.0001), 48 h (*p* value ≤ 0.0001) and 72 h (*p* value ≤ 0.0001), as compared to the control group (Fig. 3).

There was a statistically significant positive correlation between the resistin concentrations of the multiple trauma patients at time point 24 h and the corresponding SIRS score on day 2 with an *r* = 0.5969 (*p* value = 0.0342). In addition, there was a statistically significant positive correlation between the resistin concentrations of the multiple trauma patients at 48 h and the corresponding SIRS score on day 3 with an *r* = 0.5935 (*p* value = 0.0455). Furthermore, there was a statistically significant positive correlation between the resistin concentrations of the multiple trauma patients at time point 0 h and the corresponding MOF score on day 2 with an *r* = 0.7283 *(p* value = 0.0152).

### Interleukin-17A

**Fig. 4:**
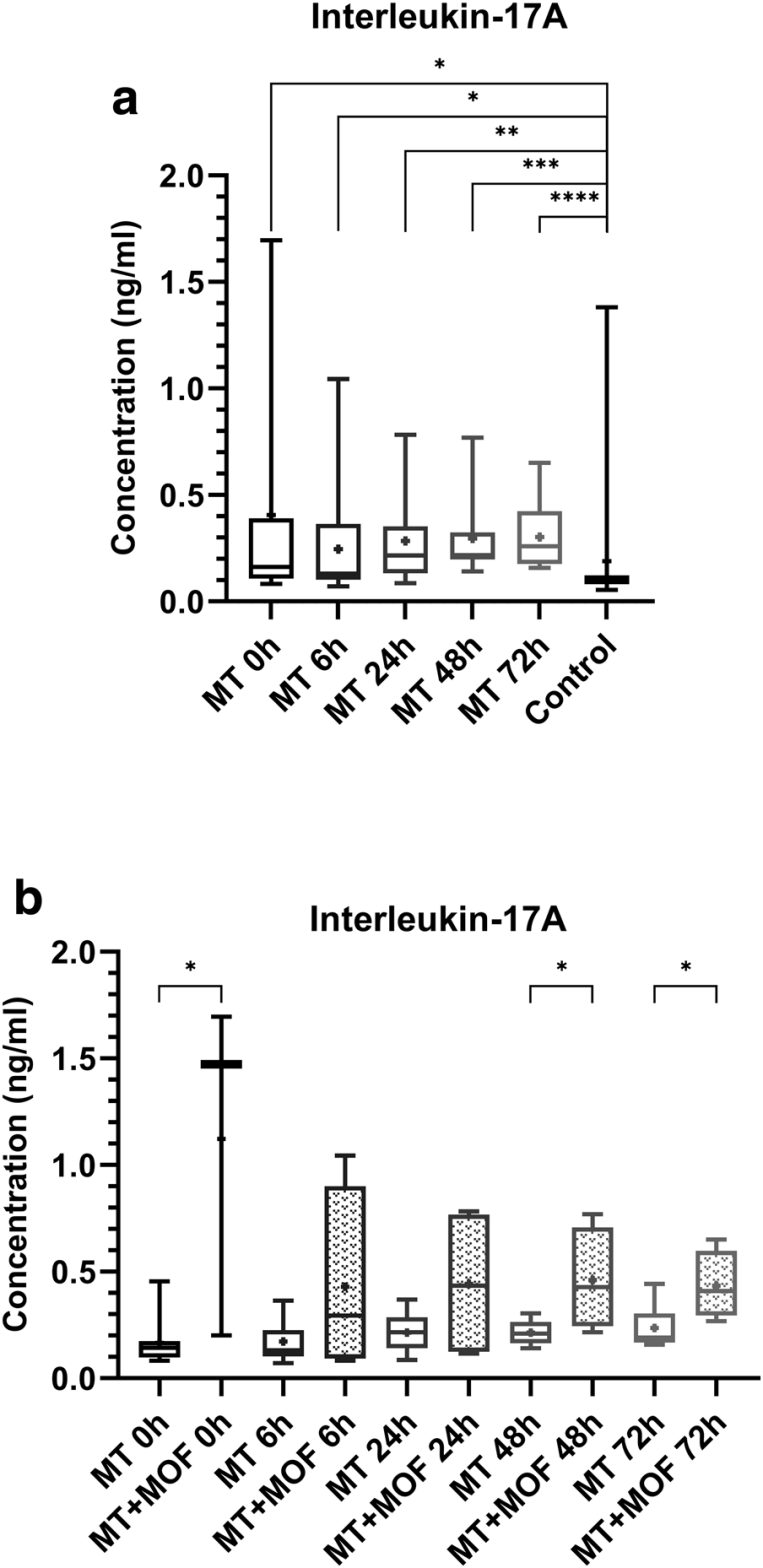
**a **Comparison of interleukin-17A plasma concentrations at the individual time points between multiple trauma patients (MT) and control group (control). Depiction of the concentrations as box-and-whisker plots with minimum/maximum value and + depicting the mean value. Statistical analysis of the data was performed by Mann–Whitney *U* test (* = *p* < 0.05; ** = *p* < 0.01; *** = *p* < 0.001; **** = *p* ≤ 0.0001). n_multiple trauma 0 h = 12_, n_multiple trauma 6 h = 14_, n_multiple trauma 24 h = 13_, n_multiple trauma 48 h = 12_, n_multiple trauma 72 h = 12_, n_control group = 14_. **b** Comparison of interleukin-17A plasma concentrations at the individual time points between multiple trauma patients without (MT) and multiple trauma patients with multiple organ failure (MT + MOF). Depiction of the concentrations as box-and-whisker plots with minimum/maximum value and + depicting the mean value. Statistical analysis of the data was performed by Mann–Whitney *U* test (* =* p* < 0.05). n_MT 0 h = 9_, n_MT + MOF 0 h = 3_, n_MT 6 h = 10_, n_MT + MOF 6 h = 4_, n_MT 24 h = 9_, n_MT + MOF 24 h = 4_, n_MT 48 h = 8_, n_MT + MOF 48 h = 4_, n_MT 72 h = 8_, n_MT + MOF 72 h = 4_

The interleukin-17A plasma concentrations of the multiple trauma patients were statistically significant higher at time point 0 h (*p* value = 0.0103), 6 h (*p* value = 0.034), 24 h (*p* value = 0.0016), 48 h (*p* value ≤ 0.0001) and 72 h (*p* value ≤ 0.0001), as compared to the control group (Fig. 4a). The multiple trauma patients with MOF showed statistically significant higher interleukin-17A concentrations at time point 0 h (*p* value = 0.0182), 48 h (*p* value = 0.0485) and time point 72 h (*p* value = 0.0238), as compared to the multiple trauma patients without MOF (Fig. 4b).

**Fig. 5:**
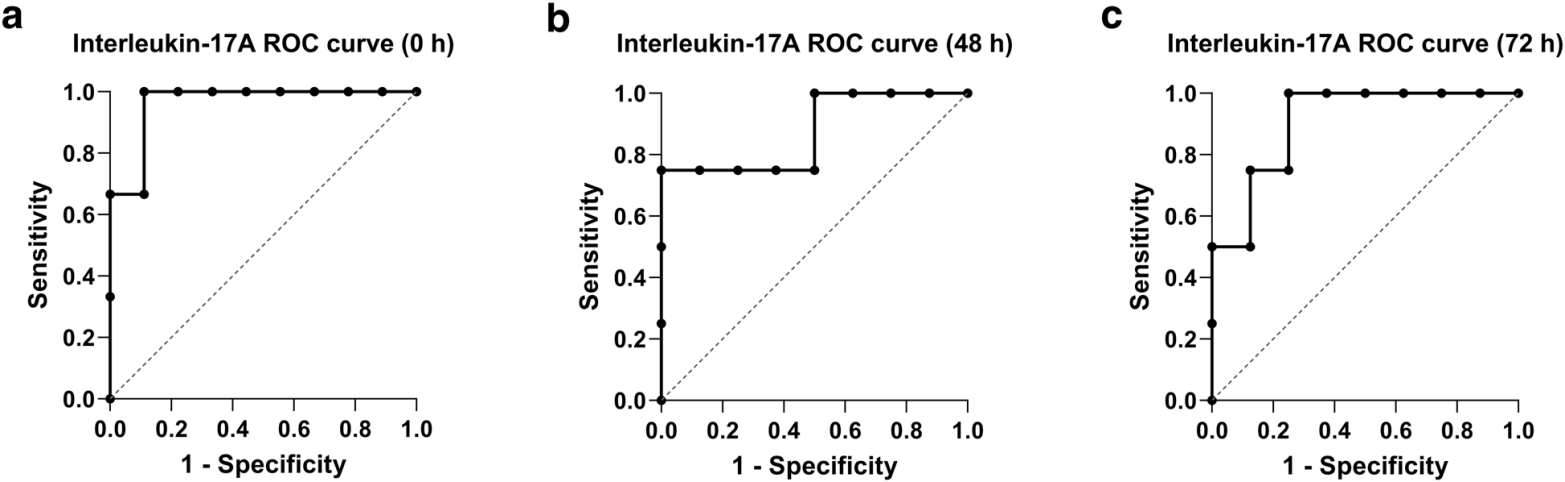
**a **Receiver operating characteristic curve (ROC curve) analysis of interleukin-17A plasma concentration at time point 0 h for the discrimination between multiple trauma patients with and without multiple organ failure (AUC = 0.9630; 95% CI  0.8566–1.00; p = 0.0208). **b** Receiver operating characteristic curve (ROC curve) analysis of interleukin-17A plasma concentration at time point 48 h for the discrimination between multiple trauma patients with and without multiple organ failure (AUC = 0.8750; 95% CI 0.6339–1.00; *p* = 0.0415). **c** Receiver operating characteristic curve (ROC curve) analysis of interleukin-17A plasma concentration at time point 72 h for the discrimination between multiple trauma patients with and without multiple organ failure (AUC = 0.9063; 95% CI 0.7327–1.00; *p* = 0.0272)

There was a statistically significant positive correlation between the interleukin-17A concentrations of the multiple trauma patients at time point 24 h and the corresponding SIRS scores on day 2 with an *r* = 0.577 (*p* value = 0.0418) and on day 3 with an *r* = 0.5935 (*p* value = 0.0455). In addition, there was a statistically significant positive correlation between the interleukin-17A concentrations of the multiple trauma patients at time point 6 h with an *r* = 0.7341 (*p* value = 0.010), 24 h with an *r* = 0.6478 (*p* value = 0.0292) and 48 h with an *r* = 0.6262 (*p* value = 0.0363) and the corresponding SIRS score on day 4. Furthermore, there was a statistically significant positive correlation between the interleukin-17A concentrations of the multiple trauma patients at time point 48 h and the corresponding MOF score on day 2 with an *r* = 0.6199 (*p* value = 0.0356). The interleukin-17A concentration showed at time point 0 h an AUC of 0.9630 (95% CI 0.8566–1.00) and a statistical significance with a *p* value of 0.0208, at 48 h an AUC of 0.875 (95% CI 0.6339–1.00) and a statistical significance with a *p* value of 0.0415 and at time point 72 h an AUC of 0.9063 (95% CI 0.7327–1.00) and a statistical significance with a *p* value of 0.0272 (Fig. 5a–c).

### Interleukin-33

**Fig. 6:**
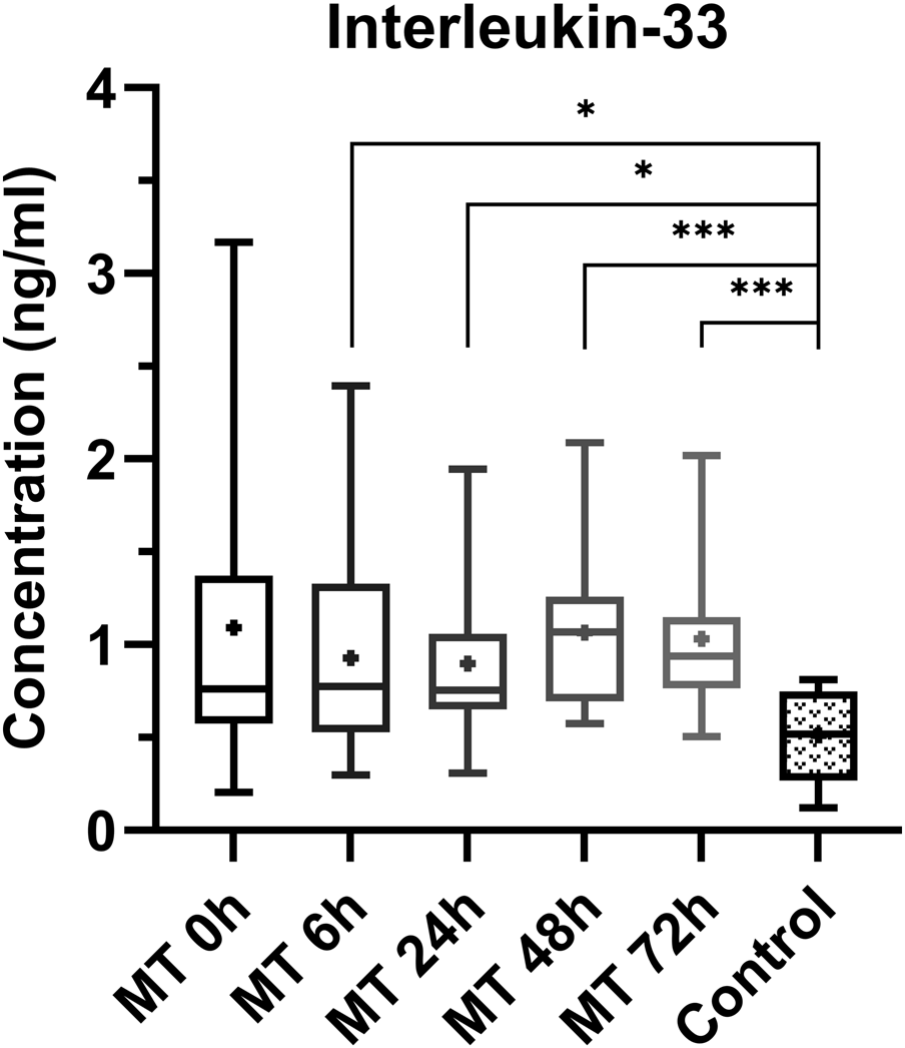
Comparison of interleukin-33 plasma concentrations at the individual time points between multiple trauma patients (MT) and control group (control). Depiction of the concentrations as box-and-whisker plots with minimum/maximum value and + depicting the mean value. Statistical analysis of the data was performed by Mann–Whitney *U* test (* = *p* < 0.05; *** = *p* < 0.001). n_multiple trauma 0 h = 12_, n_multiple trauma 6 h = 14_, n_multiple trauma 24 h = 13_, n_multiple trauma 48 h = 12_, n_multiple trauma 72 h = 12_, n_control group = 14_

The interleukin-33 plasma concentrations of the multiple trauma patients were statistically significant higher at time point 6 h (*p* value = 0.0319), 24 h (*p* value = 0.0125), 48 h (*p* value = 0.0007) and 72 h (*p* value = 0.0003) compared to the control group (Fig. 6).

There was a statistically significant positive correlation between the ISS and the corresponding interleukin-33 concentrations of the multiple trauma patients at time point 0 h with an *r* = 0.7425 (*p* value = 0.0074). Furthermore, there was a statistically significant positive correlation between the interleukin-33 concentrations of the multiple trauma patients at time point 0 h with an *r* = 0.6691 (*p* value = 0.0390), 24 h with an *r* = 0.6188 (*p* value = 0.0355) and 48 h with an *r* = 0.6622 (*p* value = 0.0223) and the corresponding SIRS score on day 3. In addition, there was a statistically significant positive correlation between the interleukin-33 concentrations of the multiple trauma patients at time point 48 h with an *r *= 0.6478 (*p* value = 0.0292) and 72 h with an *r* = 0.6262 (*p* value = 0.0363) and the corresponding SIRS score on day 4. Finally, there was a statistically significant positive correlation between the interleukin-33 concentrations of the multiple trauma patients at time point 0 h and the corresponding MOF score on day 3 with an *r = *0.7967 (*p* value = 0.0089).

## Discussion

### Patients and methods

The multiple trauma patient population of this study consisted of 57.1% men and 42.9% women, who had an average age of 49.7 years, while the patient population of the German TraumaRegister in the years 2012–2014 consisted of 68% men and 32% women, who had an average age of 57.0 years [37]. It was shown in both groups that the male sex prevails in the multiple trauma patient population, while our multiple trauma patients were younger on average. The multiple trauma patients of this study had a higher ISS with a median of 41, as compared to the TraumaRegister patients with an ISS of 29.1, who were thus more seriously injured [37]. In our study, 28.6% of the patients developed a MOF, which was comparable to the 32.7% in the TraumaRegister population between the years 2002 and 2011 [38]. In this work, the Denver MOF score was used, since Grotz and coworkers showed in their work that the Denver score has a higher specificity (88%) for the diagnosis of MOF than the Marshall score (75%) and the Goris score (78%) [39]. In addition, Hutchings and coworkers also recommend using the Denver MOF score as the gold standard for the diagnosis of multiple organ failure after trauma [40].

### Leptin

In this study, the multiple trauma patients showed statistically significant higher leptin plasma concentrations at all five time points, as compared to the control group. In the study of Chachkhiani and coworkers, the patients who experienced injury in the form of visceral surgery, also had statistically significant increased plasma concentrations of leptin at time point 24 h, as compared to the preoperative values and the healthy control group [41]. In this study, the multiple trauma patients with MOF showed higher leptin concentrations at all time points, as compared to the multiple trauma patients who did not develop MOF. In their study, Kimura and coworkers could show that patients who developed organ failure after a liver resection had significant higher leptin concentrations [42]. The leptin concentration at time point 72 h was—according to the ROC curve analysis—a very good diagnostic marker for the discrimination between multiple trauma patients who developed multiple organ failure and the patients who did not. In the work of Yousef et al., leptin turned out to be a highly sensitive and specific marker for the discrimination between patients with SIRS or sepsis and patients without [43]. However, it should not be disregarded that other factors besides trauma have an influence on the leptin concentration. Thus, there is a significant correlation between the basal leptin concentration before surgery, which in turn correlates with BMI and peak leptin levels after surgery [44].

### Resistin

The multiple trauma patients in this study showed statistically significant higher resistin plasma concentrations at all five time points, as compared to the control group. Dong et al. revealed that patients with an isolated traumatic brain injury show significant increased resistin plasma concentrations within the first 7 days after trauma [45]. In our study, there were statistically significant positive correlations between the resistin concentrations of the multiple trauma patients and the corresponding SIRS and MOF scores. Wade et al. found a significant positive correlation between the resistin plasma concentrations and the multiple organ dysfunction score in patients with burn trauma [46]. In addition, Sundén-Cullberg et al. demonstrated a statistically significant correlation between the resistin concentrations and the SOFA score in sepsis patients [47].

### Interleukin-17A

The multiple trauma patients showed statistically significant higher interleukin-17A plasma concentrations throughout the observation period, as compared to the control group. This is in line with the work of Abboud et al., who highlighted the importance of IL-17A in the post-traumatic immune response in patients with a severe blunt trauma [48]. Furthermore, Hefele et al. discovered an increased IL-17A expression on TH-17 cells and CD4 + Tregs following trauma [49]. In this study, the multiple trauma patients with MOF showed statistically significant higher IL-17A concentrations at the time points 0 h, 48 h and 72 h, as compared to the trauma patients without MOF. With the concordant findings of Abboud et al., IL-17A may play a role in the development of MOF [48]. In our study, there were statistically significant positive correlations between the IL-17A concentrations of the multiple trauma patients and the corresponding SIRS and MOF scores. In another study, however, no association was found between IL-17A levels and the multiple organ dysfunction score in multiple trauma patients [50]. This may be explained by the fact that in that study only IL-17A concentrations at the day of admission to the intensive care unit were examined. In our study, the correlation between IL-17A and the MOF score was observed at 48 h. According to the ROC curve analysis, the IL-17A concentrations at time points 0 h, 48 h and 72 h were very good diagnostic markers for the discrimination between multiple trauma patients who developed multiple organ failure and patients who did not. In the above discussed study from Abdelkader et al. it could be shown that multiple trauma patients with sepsis had elevated IL-17A concentrations and that IL-17A is a possible predictor of sepsis with an AUC of 0.687 [50].

### Interleukin-33

The multiple trauma patients showed higher interleukin-33 concentrations at all five time points and at 6 h, 24 h, 48 h and 72 h statistically significant higher concentrations, as compared to the control group. Furthermore, there was a statistically significant positive correlation between the ISS and the corresponding IL-33 concentrations of the multiple trauma patients at time point 0 h. These results are consistent with the function of IL-33 as an alarmin, which is released after cell damage in the context of multiple trauma [51]. Another study could demonstrate that trauma patients had elevated IL-33 concentrations in the first 7 days after severe blunt trauma [52]. However, there was no correlation between the IL-33 concentrations and the ISS [52]. This could be caused by the fact that these trauma patients were less seriously injured with a mean ISS of only 20.2 and the IL-33 concentration possibly shows a stronger correlation with higher ISS values. In our study, there were statistically significant positive correlations between the IL-33 concentrations of the multiple trauma patients and the corresponding SIRS and MOF scores. Xu et al. could demonstrate that trauma patients with an organ failure show elevated IL-33 levels [52].

## Conclusion

The four adipokines investigated may play a role in the early posttraumatic immune response and the adipokines leptin, IL-17A and IL-33 in the development of SIRS and MODS. In addition, leptin and IL-17A may be useful as diagnostic biomarkers to identify multiple trauma patients at risk of developing multiple organ failure in an intensive care setting.

The insights gained in this study can help to identify patients with a developing multiple organ failure early within the framework of systemic inflammatory response syndrome after severe trauma, potentially leading to better treatment outcomes. Furthermore—as part of the Damage Control Surgery concept—the information of the adipokine concentrations could be included in the treatment planning. In this way, the decision on the invasiveness of the primary trauma care (damage control orthopedics vs. early total care) in risk patients can be made more profoundly to avoid a possible second hit. In addition, the adipokine levels could be used, not only for diagnosis and therapy planning, but also for monitoring the success of surgical and intensive care. Lastly, the insights found in this study now need to be confirmed and validated in a multicenter study with a significant larger number of multiple trauma patients.

## Data Availability

The datasets used and/or analysed during the current study are available from the corresponding author on reasonable request.

## Abbreviations

AIS: Abbreviated injury scale
AUC: Area under the curve
BMI: Body mass index
C: Celsius
CI: Confidence interval
ELISA: Enzyme-linked immunosorbent assay
EDTA: Ethylenediaminetetraacetic acid
g: Gravity
ISS: Injury severity score
h: Hour
IL: Interleukin
μl: Microliter
ml: Milliliter
mm^3^: Cubic millimeter
MODS: Multiple organ dysfunction syndrome
MOF: Multiple organ failure
MT: Multiple trauma
NF: Nuclear factor
OD: Optical density
ROC: Receiver operating characteristic
SD: Standard deviation
SIRS: Systemic inflammatory response syndrome
TGF: Transforming growth factor
TH: T helper
TNF: Tumor necrosis factor
